# Molecular System Bioenergics of the Heart: Experimental Studies of Metabolic Compartmentation and Energy Fluxes *versus* Computer Modeling †

**DOI:** 10.3390/ijms12129296

**Published:** 2011-12-13

**Authors:** Mayis Aliev, Rita Guzun, Minna Karu-Varikmaa, Tuuli Kaambre, Theo Wallimann, Valdur Saks

**Affiliations:** 1 Institute of Experimental Cardiology, Cardiology Research Center, Moscow, 121552, Russia; E-Mail: mayis_aliev@yahoo.com; 2 INSERM U1055, Laboratory of Fundamental et Applied Bioenergetics, Joseph Fourier University, 2280 Rue de la Piscine, BP 53, Grenoble Cedex 9, France; E-Mail: rita.guzun@gmail.com; 3 Laboratory of Bioenergetics, National Institute of Chemical Physics and Biophysics, Akadeemia tee 23, 12618 Tallinn, Estonia; E-Mails: minna.karu@kbfi.ee (M.K.-V.); tuuli.kaambre@kbfi.ee (T.K.); 4 Professor emeritus, formerly at Institute of Cell Biology ETH Zurich; Present Address: Schuermattstrasse 23 CH-8962 Bergdietikon, AG Switzerland; E-Mail: theo.wallimann@cell.biol.ethz.ch

**Keywords:** heart, respiration, energy transfer, phosphocreatine, mathematical modeling

## Abstract

In this review we analyze the recent important and remarkable advancements in studies of compartmentation of adenine nucleotides in muscle cells due to their binding to macromolecular complexes and cellular structures, which results in non-equilibrium steady state of the creatine kinase reaction. We discuss the problems of measuring the energy fluxes between different cellular compartments and their simulation by using different computer models. Energy flux determinations by ^18^O transfer method have shown that in heart about 80% of energy is carried out of mitochondrial intermembrane space into cytoplasm by phosphocreatine fluxes generated by mitochondrial creatine kinase from adenosine triphosphate (ATP), produced by ATP Synthasome. We have applied the mathematical model of compartmentalized energy transfer for analysis of experimental data on the dependence of oxygen consumption rate on heart workload in isolated working heart reported by Williamson *et al*. The analysis of these data show that even at the maximal workloads and respiration rates, equal to 174 μmol O_2_ per min per g dry weight, phosphocreatine flux, and not ATP, carries about 80–85% percent of energy needed out of mitochondria into the cytosol. We analyze also the reasons of failures of several computer models published in the literature to correctly describe the experimental data.

## 1. Introduction

December 17, 2010 the Journal of Biological Chemistry published a long-awaited breakthrough article which is a decisive step in the research of muscle cell energetics. This is the article by Christine Nabuurs, Bertolt Huijbregts, Be Wieringa, Cees W. Hilbers and Arend Heerschap from the University of Nijmegen, The Netherlands, entitled “^31^P Saturation Transfer Spectroscopy Predicts Differential Intracellular Macromolecular Association of ATP and ADP in Skeletal Muscle” [1]. In this important work the authors reported the experimental results, for the first time directly showing that adenosine triphosphate (ATP) and adenosine diphosphate (ADP) in muscle cells are bound to macromolecules and that this results in a non-equilibrium state of the creatine kinase (CK) reaction [1]. These data contribute significantly to the explanation of the cellular mechanisms of ATP compartmentation [2,3]. Equally important and consistent with this work are the series of publications by Dzeja, Terzic and Ingwall [4–9], reporting direct measurements of the energy fluxes in cardiac cells *in vivo* by ^18^O transfer method in health and in pathology. These works logically develop and experimentally demonstrate the validity of the concepts of compartmentalized energy metabolism that were developed over the last 40 years of research in many laboratories, including those of the authors of this review [9–26]. These works significantly helped the formulation of the principles of Molecular System Bioenergetics [27]. Our aim, here, is to comment on the current state of research of muscle energy metabolism in a historical perspective, and also to analyze in details some published contradicting results, relating to the question of whether energy transfer in muscle is based on compartmentalized and vectorial processes at the subcellular level or whether the substrates in a muscle cell behaves in full equilibrium as in “a bag with enzymes in solution” [2,28]. The latter was considered in a number of publications based on computer modeling of whole energy metabolism [2]. In our opinion, the reasons for the failures by some laboratories to detect the compartmentalized energy transfer are mostly related to simplified assumption for computer modeling of these highly complex processes (for reviews see [2,28]) or to choosing non-suitable experimental models and set-ups to answer this question (see the last section of this review).

### 1.1. Some Historical Notes on the Metabolic Compartmentation of Adenine Nucleotides in Muscle Cells

In the research of energy metabolism of muscle cells, including heart, the problem of compartmentation of adenine nucleotides in the cells is intimately related to the very role of the creatine kinase (CK) system. The role of the creatine kinase system in muscle energetics and relative contributions of ATP and PCr into energy supply for contraction have been actively debated for more than half a century. The subject has a very interesting history, full of contradictions, but at present a valid solutions at a quantitative level can be proposed for the elucidation of this complex and intriguing problem.

After discoveries of phosphocreatine (PCr) in 1927 and ATP in 1929, Lundsgaard described the relationship between contractile force and PCr content in muscles with inhibited glycolytic lactate production, and Lohman discovered in 1934 the creatine kinase (CK) reaction [29–33]. In his famous article entitled “Revolution in Physiology” published in Physiological Review in 1932, Hill acknowledged the end of popular at that time “lactate theory of contraction” and emphasized the role of phosphorous compounds in muscle energetics [34]. In 1939 Belitzer and Tsybakova showed that creatine (Cr) added to muscle homogenate stimulated (without addition of ADP) respiration and phosphocreatine (PCr) production with PCr/O_2_ ratio between 5.2 and 7 [35,36]. In 1950, Hill had to write another review with his famous challenge to biochemists to find convincing evidence whether ATP or PCr was the immediate supplier of energy for contraction [37], since in physiological experiments, with rapid sampling of tissue, only a decrease of PCr was seen during the contraction cycle, but experiments with the actomyosin system showed that contraction needs ATP [33,37]. In 1962–1965 Davies *et al*., showed by inhibiting CK with 2,4-dinitrofluorobenzene that under these conditions ATP is used during contraction [38]. This established the central role of ATP in muscle energetics, and PCr was given the rather modest role of a simple energy store used to replenish ATP at increased workloads where the CK reaction was *a priori* taken to be in rapid equilibrium [33]. Very rapidly, it became evident that this simple and convenient theory (still popular among many authors as critically reviewed in [2]) is not consistent with a plethora of important experimental observations. Among these, important data came from studies of muscle pathologies. If there were a direct transfer of ATP from mitochondria to myofibrils and to ATPases at diverse subcellular locations with high affinity to ATP, one might expect that normal cardiac function should be maintained until complete exhaustion of ATP. This, however, is never observed: Gerken and Schlette showed already in 1968 that inhibition of creatine kinase with 2,4-dinitrofluorobenzene results in heart failure even though 80–85% of cellular ATP were still present in the cells [39]. Gudbjarnason *et al*. [40] and Neely *et al*. [41] published first the results of detailed studies of metabolic changes in ischemic heart, showing that, in the absence of mitochondrial oxidative phosphorylation, cardiac contraction stops in the presence of approximately still 90% of cellular ATP after almost complete utilization of phosphocreatine. Kammermeyer *et al*. showed that cardiac contraction decreases in hypoxic hearts at unchanged phosphorylation potential values and is thus a result of changes in kinetic but not thermodynamic factors [42]. Neely and Grotyohann, and also Kupriyanov *et al*. [43–45] showed that about 70% of cellular ATP can be removed by perfusion of the hearts by desoxyglucose. This latter compound is phosphorylated in the hexokinase reaction but the desoxyglucose-6-phosphate formed is not metabolized further, thus acting as a phosphate trap—the ADP produced is converted into adenosine monophosphate (AMP) by the myokinase reaction and into adenosine by 5′-nucleotidase, with adenosine, which is cell permeable, easily leaving the cell [46], without any changes in the contractile force if creatine and phosphocreatine are present at normal concentrations. All these results were interpreted as showing ATP compartmentation in the cells and initiated a host of very intensive studies on the role of creatine kinase and other phosphotransfer systems that eventually led to the description of the phosphocreatine pathway (shuttle or circuit) of energy transfer in cardiac cells connecting different ATP compartments [2,3]. This pathway is described in detail elsewhere [8–28] and plays an important role in feedback regulation of respiration by cardiac work [8,19,23,24].

### 1.2. Local Restrictions of ATP and ADP Diffusion, Their Binding to the Intracellular Structures and on the State of the Creatine Kinase Reaction in Muscle Cells

**Figure 1:**
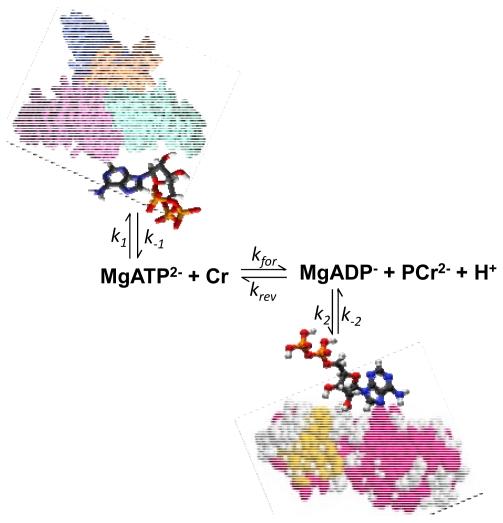
Illustration of the non-equilibrium state of the creatine kinase reaction in muscle cells due to ATP and ADP binding to the proteins and solid biological structures. Data from Nabuurs *et al*. [1].

^31^P saturation transfer spectroscopy has been intensively used to study the kinetics of the creatine kinase reaction in muscle cells by saturating γ-ATP phosphate and recording the transfer of magnetization to PCr [47–50]. Nabuurs *et al*. used this method to study the kinetics of the phosphoryl exchange involving ATP and ADP in the muscles of normal mice and in those lacking the cytosolic creatine kinase and adenylate kinase isoforms [1]. They observed also a decrease of the β-ATP signal upon saturation of the γ-ATP phosphate in both types of tissues as nuclear Overhauser effects, and detailed analysis of this phenomenon showed that this can be explained as a result of exchange between free cellular ATP and ATP bound to slowly rotating macromolecules [1]. The second most important conclusion in this work was that free ADP is only transiently present in the cytosol due to ADP binding to solid-like structures [1]. That means that the creatine kinase cannot be in equilibrium, and that this reaction in the cells is better presented by a scheme as shown in the Figure 1. According to this scheme, a steady state is maintained between free MgADP conversion in the CK reaction (rate constants k_for_ and k_rev_) and its binding (rate constants k_2_ and k_−2_).

**Figure 2:**
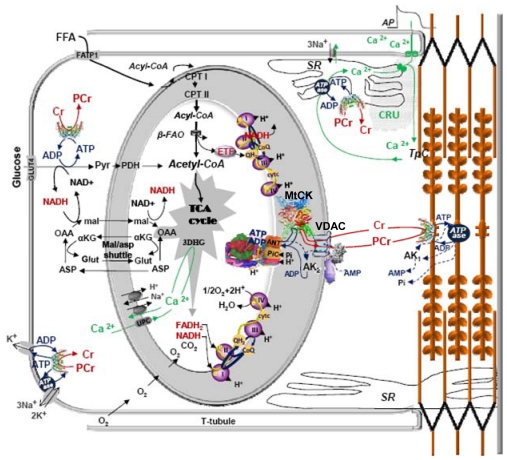
Functional scheme of the Intracellular Energetic Units of adult cardiac muscle cell.

Remarkably, this may be true not only for the cytoplasmic compartment, but probably even more so for other compartments of ATP and the subcellular localization of the creatine kinase isoforms in cardiac cells (Figure 2). This figure describes the localization of creatine kinase isoenzymes within the Intracellular Energetic Units (macrocompartments) formed by mitochondria and adjacent ATPases [18,24]. MM-creatine kinase localized in myofibrils at M-line [51] and in I-band of sarcomeres [52] rephosphorylates ADP released from active center of myosin ATPase within contraction cycle [53]. This is necessary to release the actomyosin ATPase reaction from product inhibition by ADP, a structural analog of ATP [53]. Another part of MM-creatine kinase is associated with sarcolemma [54,55] and with the membrane of sarcoplasmic reticulum [56] to regenerate continuously the local pools of ATP for membrane ATPases and for inhibition of ATP dependent K-channel of sarcolemma [57].

Free fatty acids (FFA) taken up by a family of plasma membrane proteins (FATP1), are esterified to acyl-CoA which further enter the β-fatty acids oxidation (β-FAO) pathway resulting in acetyl-CoA production. CPT I and CPT II—carnitine palmitoyltransferases I and II, respectively; Electron-transferring flavoprotein (ETF)-ubiquinone oxidoreductase delivers electrons from β-FAO *directly* to complex III of the respiratory chain (RC). Nicotinamide adenine dinucleotide (NADH) produced by β-FAO is oxidized in the complex I of the RC passing along two electrons and two protons which contribute to the polarization of mitochondrial inner membrane (MIM). Glucose (GLU) is taken up by glucose transporter-4 (GLUT-4) and oxidized via Embden-Meyerhof pathway. Pyruvate produced from glucose oxidation is transformed by the pyruvate dehydrogenise complex (PDH) into acetyl-CoA. The NADH redox potential resulted from glycolysis enters mitochondrial matrix via malate–aspartate shuttle. Malate generated in the cytosol enters the matrix in exchange for α-ketoglutarate (αKG) and can be used to produce matrix NADH. Matrix oxaloacetate (OAA) is returned to the cytosol by conversion to aspartate (ASP) and exchange with glutamate (Glut).

Acetyl-CoA is oxidized to CO_2_ in the tricarboxylic acids (TCA) cycle generating NADH and FADH_2_ which are further oxidized in the RC (complexes I, II) with final ATP synthesis. G6P inhibits HK decreasing the rate of glucolysis. The key system in energy transfer from mitochondria to cytoplasm is Mitochondrial Interactosome (MI). MI is a supercomplex, formed by ATP synthase, adenine nucleotides translocase (ANT), phosphate carriers (PIC), mitochondrial creatine kinase (MtCK), voltage-dependent anion channel (VDAC) with bound cytoskeleton proteins (specifically βII-tubulin). MI is responsible for the narrow coupling of ATP/ADP intramitochondrial turnover with phosphorylation of creatine (Cr) into phosphocreatine (PCr). PCr is then used to regenerate ATP locally by CK with ATPases (actomyosin ATPase, sarcoplasmic reticulum SERCA and ion pumps ATPases). The rephosphorylation of ADP in MM-CK reaction increases the Cr/PCr ratio which is transferred towards MtCK via CK/PCr shuttle. A small part of ADP issued from ATP hydrolysis creates gradient of concentration transmitted towards the matrix.

The shaded area in the upper right corner shows the Calcium Release Unit [58].

Calcium liberated from local intracellular stores during excitation-contraction coupling through calcium-induced calcium release mechanism, (1) activates contraction cycle by binding to troponin C in the troponin-tropomyosin complex of thin filaments and (2) enters the mitochondria mainly via the mitochondrial Ca^2+^ uniporter (UPC) to activate three Krebs cycle dehydrogenases: PDH, αKG, isocitrate dehydrogenase. Reproduced from [24] with modifications.

Under hypoxic conditions, when the PCr concentration decreases because of lack of oxygen for mitochondrial oxidative phosphorylation and coupled synthesis of phosphocreatine, the ATP-dependent K-channels are open and repolarize the membrane, thus terminating the contraction cycle and saving the energy for cell survival [3,57,59,60]. In spite of low Km for ATP of the K-channels, they are still open in the presence of relatively high concentrations of ATP in cytoplasm [59,60]. Studies of these phenomena [57] and also the MgATP-related reaction in myofibrils [61] have predicted that the ATP apparent diffusion coefficient is locally decreased by the factor of 10^5^ (to about 1.6 × 10^−11^ cm^2^/s instead 1.5 × 10^−6^ cm^2^/s for free diffusion of nucleotides) both in sarcomeric and subsarcolemmal spaces, thus resulting in functional compartmentation of ATP in these areas, connected with mitochondrial and cytoplasmic pools of ATP by the phosphotransfer networks [59–61]. Such a strong decrease in the apparent diffusion coefficients for adenine nucleotides is a result of their binding to proteins and may be related to the data reported by Nabuurs *et al*. [1]. The binding of ATP and ADP to proteins may involve interactions of adenine moieties of ATP and ADP with aromatic amino acids’ residues and many other mechanisms, absent in the case of creatine and phosphocreatine.

Work involving polar, elongated cells, where diffusional distances from the mitochondria to subcellular sites of ATP consumptions are long, such as in photoreceptor cells, hair bundle cells of the inner ear and spermatozoa show indeed that diffusional limitations of adenine nucleotides are overcome by the CK system, shuttling PCr and Cr instead of ATP and ADP from sites of ATP production (mitochondria and glycolysis) to sites of ATP consumption [62–65]. Most important, these data indicate that diffusion of ADP back from sites of ATP consumption towards the mitochondria is the key limiting factor for energy transfer, since under normal conditions cellular ADP concentration is kept very low at micromolar level and since this low amount of ADP is mostly bound to subcellular structures, enzymes and macromolecules, thus not available for free diffusion [1].

In good concord with the work of Nabuurs *et al*. [1] is yet another work from Nijmegen University, that by Van Deursen *et al*. from Be Wieringa’s laboratory [66]. These authors showed that in mouse mutants with decreased MM-creatine kinase activity the fluxes between PCr and ATP measured by ^31^P NMR inversion transfer technique became invisible when the creatine kinase activity was decreased to 34% of normal activity in wild-type mice [66]. The authors concluded that a possible explanation for this phenomenon might be that ^31^P NMR is only detecting the phosphoryl flux through MM-CK dimers in the sarcoplasm and not through the MM-CK fraction associated with the sarcolemma, sarcoplasmic reticulum and myofibrillar M and I bands or mitochondrial-bound MtCK [66]. This is in agreement with the data by Neely *et al*. [43] discussed above that only about 30% of cellular ATP is necessary for normal cardiac function if the creatine kinase system functions normally. On the other hand, experimental and clinical studies have shown that a decrease of the creatine and phosphocreatine contents in heart results in cardiac failure and the PCr/ATP ratio is a valuable diagnostic index of mortality rate in patients with dilated cardiomyopathy [11,67–70].

### 1.3. Direct Measurement of the Energy Fluxes in Vivo: The ^18^O Transfer Method

Because of ATP compartmentation in the muscle cells *in vivo*, it is most important to quantitatively measure the energy fluxes between different cellular compartments to understand and describe correctly the energy fluxes and feedback metabolic regulations of respiration. There is the most classical and very precise method to perform these measurements quantitatively: the isotope tracer method. In the bioenergetic studies of phosphoryl transfer, the most effective has been the use of ^18^O transfer [4–9,71–74]. This method is based on the following two reactions: ATP hydrolysis by water molecules containing ^18^O and ATP resynthesis with formation of [^18^O]γATP [72]:

ATP+[O18]H2O→[O18]Pi+ADPREACTION 1. ATP hydrolysis[O18]Pi+ADP→[O18]γATPREACTION 2. ATP synthesis

Paul Boyer has used this method for studies of the ATP synthase reaction [71–73]. Inclusion of [^18^O] Pi into [^18^O] γ-ATP in the presence of uncouplers led him to the conclusion of the rotary mechanism of the ATP synthesis and then to Stockholm to get the Nobel Price for this discovery [73]. Nelson Goldberg, Petras Dzeja and André Terzic with their coworkers have successfully developed and applied this method for studies of the kinetics of phosphoryl-transfer reactions and energy fluxes in the cells *in vivo* by measuring the rates of the following reactions 3–5 [4–8,74]:

[O18]γATP+Cr→[O18] PCr+ADPREACTION 3. Creatine kinase phosphotransfer[O18]γATP+AMP→[O18]βADP+ADP→[O18]βATP+AMPREACTION 4. Adenylate kinase phosphotransfer[O18]γATP+Glucose→[O18]G6P+ADPREACTION 5. Glycolytic phosphotransfer

If there is a direct transfer ATP from mitochondria to MgATPases and its immediate hydrolysis for contraction as sometimes proposed in the literature, only the isotope transfer reactions 1 and 2 can be observed. Instead, Dzeja’s group has shown in the excellent series of studies during several decades that in normal cardiac cells about 80–85% of phosphoryl groups and thus energy is carried out of mitochondria by the phosphocreatine flux, and about 10–15% of energy by adenylate kinase system, with minor importance of the glycolytic phosphotransfer network [4–9]. These fluxes increase linearly with the increase of the heart workload under conditions of the Frank-Starling law [9]. The role of adenylate kinase system increases significantly in hypoxia and heart pathology [4].

**Figure 3:**
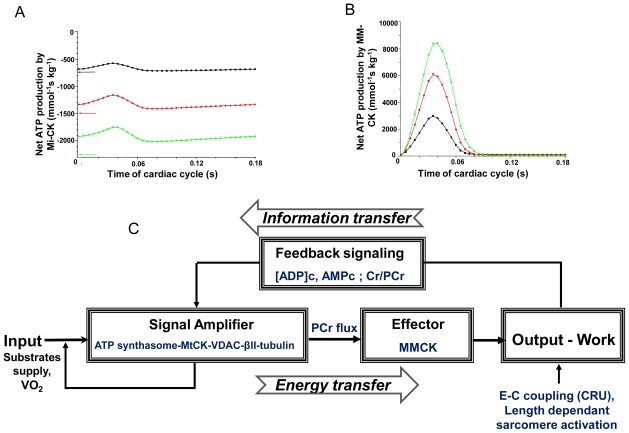
General presentation of the feedback metabolic signaling in regulation of energy metabolism within Intracellular Energetic Units in cardiac cells. (**A**) Workload dependence of the dynamics of net PCr production by MtCK within cardiac contraction cycle; (**B**) Workload dependence of the dynamics of net ATP production by MM-CK in myofibrils within cardiac contraction cycle. The average cardiac cycle time is 180 milliseconds. Workload values are indicated by respective color. The reaction rates for workloads of 750 (black), 1500 (red) and 2250 (green) μmol ATP s^−1^ kg^−1^ are shown. For calculations, complete model of Aliev, Saks and Dos Santos described below was used. In mitochondria (**A**), due to the functional coupling between ANT and MtCK, the reaction runs always out of equilibrium in the direction of PCr synthesis, the steady-state values of the rates of this coupled reactions are increased by increasing the workload which induce cyclic changes of MgATP and MM-CK reactions in myofibrils (**B**); (**C**) Due to the non-equilibrium steady-state MtCK (**A**) and non-equilibrium cyclic MM-CK reactions (**B**) intracellular ATP utilization (marked as output) and mitochondrial ATP regeneration (marked as input) are interconnected via the cyclic fluctuations of cytosolic ADP, AMP and Cr/PCr (**C**). For explanation see the text. Adapted from [19] with modifications; Due to the non-equilibrium steady-state MtCK (**A**) and non-equilibrium cyclic MMCK reactions (**B**) intracellular ATP utilization (marked as output) and mitochondrial ATP regeneration (marked as input) are interconnected via the cyclic fluctuations of cytosolic ADP, AMP and Cr/PCr (**C**). For explanation see the text. Adapted from [19].

These data are in excellent concord with the results of biochemical studies on the role of compartmentalized creatine kinase in muscle cells carried out in many laboratories during several decades [9–28]. Our recent studies have shown that in heart mitochondria the voltage-dependent anion channel (VDAC) in the outer membrane is controlled by cytoskeletal proteins including tubulin beta II that resulting in selective restriction of the permeability for adenine nucleotides but not for creatine or phosphocreatine [18,20–24,75]. It has been proposed that the structure responsible for effective synthesis of phosphocreatine in the muscle and brain cells is the Mitochondrial Interactosome (MI)—A supercomplex consisting of ATP synthasome, mitochondrial creatine kinase, VDAC and probably beta II tubulin [18,75]. Within this supercomplex, the continuous recycling of adenine nucleotides is coupled to effective synthesis of the phosphocreatine [18,19,75]. Application of the methods of Metabolic Control Analysis for studies of functioning of MI has shown that this supercomplex is very effective amplifier of the metabolic signals from cytoplasm [76,77]. Figure 3 shows the Scheme of energy fluxes and metabolic feedback regulation of respiration in cardiac cells based on mass and energy transfer by PCr from mitochondria into cytoplasm and information transfer by cyclic changes of PCr, Cr, ADP and AMP within each contraction cycle. Direct measurements both *in vivo* [4–9] and in permeabilized cardiomyocytes *in situ* [75] show that the main energy flux is carried out into cytoplasm by PCr molecules with PCr/O_2_ ratio about 5.6 [9,75], in good agreement with the results of simulations by model of compartmentalized energy transfer (see below). Within contraction cycle actomyosin ATPase reaction produces MgADP, which is rapidly rephosphorylated by MM-CK, resulting in ADP concentration transients depending on workload (Figure 3B). Simultaneous changes in [PCr]/[Cr] ratio within contraction cycle together with ADP and AMP transients represent metabolic feedback signals which are strongly amplified in coupled reactions within MI. This scheme has been described in details before [18,19], here we have added the indication of a possible role for adenylate kinase in this beat to beat metabolic feedback regulation of respiration, in accordance with the experimental data by Dzeja *et al*. [4].

The mechanisms of functioning of the Mitochondrial Interactosome and other coupled reactions in complex phosphotransfer pathways are best explained by the theory of vectorial metabolism and the vectorial ligand conduction, proposed by P. Mitchell, 1979 [78]. This theory corresponds well to the ever increasing number of experimental data showing that in living systems proteins function in a concentrated and complicated environments [79] within organized metabolic dissipative structures [80–82] and metabolic networks [4]. Vectorial metabolism by ligand conduction within multienzyme complexes allows overcoming the diffusion problems for metabolites including ATP, part of which has been found to be associated with proteins in muscle cells [2,3,17,21,83].

Decisive role in organization of metabolic pathways of vectorial ligand conduction, in particular in formation of ICEUs belongs to cytoskeleton and to its interaction with mitochondria [17–22,84]. Most interestingly, due to ATP compartmentation in the sites of its utilization, the creatine kinase system as an energy supplier plays important role in maintaining the cytoskeletal structures and thus the specific cellular structural organization [85].

### 1.4. Problems of Computer Simulation of Muscle Energetic: Success and Failures

**Figure 4:**
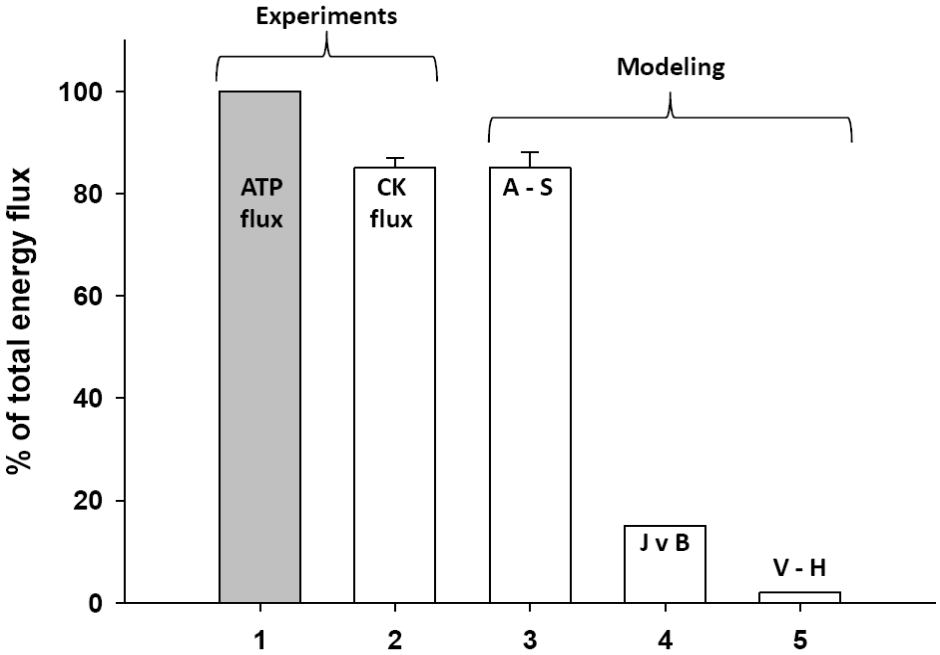
Comparison of the experimental data of the energy flux measurements with the results of simulations by different mathematical models. ATP flux—The rate of ATP synthesis in mitochondria [4–8]; CK flux—Energy flux carried into cytoplasm by phosphocreatine measured experimentally by ^18^ O transfer method [4–8]; A-S: Aliev and Saks models of compartmentalized energy transfer [88,89]; JvB—Calculation of the CK fluxes in the heart by Hetting and van Beek [90]. No fitting with the experimental data. V-H—Determination of CK fluxes in the heart by Vendelin and Hoerter [91]. The CK fluxes disappeared at medium level workloads due to necrosis induced by isoproterenol and high Ca^2+^ concentration in perfusate.

In the studies of complex metabolic networks, as phosphotransfer reactions described in Figure 2, and in the Systems Biology in general, the computer simulation is an important and effective method of investigation [86]. The most important requirement initially pointed out already by Claude Bernard some 150 years ago is that computer analysis should be based on reliable experiments and the results of modeling should fit independent experimental data [87]. There are very reliable data collected in many experimental studies of energy fluxes by Dzeja *et al*. described above [4–8]. Figure 4 shows comparison of these experimental data with the results of simulations by three different models of functioning of the creatine kinase in heart cells: the model of compartmentalized energy transfer developed by Aliev and Saks (A-S model) [88,89], the multiscale “sloppy” modeling by Johannes van Beek group (JvB model) [90], and the results of Vendelin-Hoerter study (V-H model) [91].

Only the first model gives a good fitting with the experimental data, showing that about 85% of energy produced in mitochondria as ATP flux is carried out of mitochondria as PCr flux [88,89], in concord with the multiple experimental data reported by Dzeja *et al*. [4–8]. The model described by Hettling and van Beek does not agree with the experimental data, giving for PCr flux only about 15% of total energy flux into the cytoplasm [90], and Vendelin–Hoerter group has completely failed to detect any PCr flux at increased workloads [91]. How can we explain these discrepancies? Given below are short analyses of these models to understand the shortcomings of these two last models and thus to find out a valid way of construction of the meaningful models needed.

#### 1.4.1. Original and Modified Models of Compartmentalized Energy Transfer

**Figure 5:**
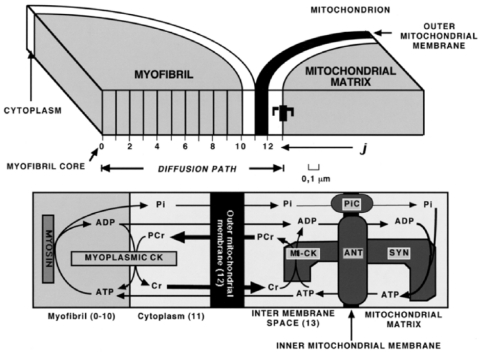
The general scheme of compartmentalized energy transfer in cardiac cell.

The model of Aliev and Saks, first developed in 1996 [92] and in a more detailed version in 1997 [89], considers the time-dependent diffusional exchange of ATP, ADP, PCr, Cr and Pi between myofibrils and intramyofibrillar mitochondria along their radii and a thin layer of cytoplasm interposed among them in cardiomyocytes (Figure 5). Metabolite levels along this diffusion path are determined by the interplay of ATP consuming and restoring reactions in cardiac cell, transport of ATP and ADP across the mitochondrial membranes, phosphotransfer reactions and diffusion.

The model [88,89] considers diffusional exchange of ATP, ADP, PCr, Cr and Pi between myofibrils and mitochondria along their radii and an interposed among them a layer of cytoplasm. Diffusion path of 1.3 μm length includes ten 0.1 μm space units (J) in myofibril and 3 units in cytoplasm, mitochondrial outer membrane and intermembrane compartments.

Lower part of figure shows compartmentation of CK in myofibril and cytoplasm spaces (Myoplasmic CK) and mitochondrial intermembrane space (MtCK). Mitochondrial ATP synthase (Syn) localises in mitochondrial matrix space; mitochondrial adenine nucleotide translocase (ANT) and Pi carrier (PiC) are in mitochondrial inner membrane (Inner mitochondrial membrane). Myofibrillar myosin provides ATP hydrolysis during myofibril contraction. MtCK and ANT are proposed to be coupled by high local ATP concentration, arising from restricted ATP diffusion in the narrow gap (microcompartment) between coupled molecules. Arrows indicate diffusion fluxes of metabolites in compartments and between them through mitochondrial outer membrane. Adapted from [88,89].

The mitochondrial block of the model is based on a kinetic scheme of mitochondrial ATP synthase with parameters allowing the description of experimental ADP and Pi dependences of oxidative phosphorylation [88], obtained in laboratories of Wilson and Chance on isolated mitochondria [93,94]. In mitochondria, the ATP/ADP translocase and the Pi carrier regulate the matrix concentrations of ATP, ADP and Pi available for the ATP synthase activation. These carriers establish constant positive ADP and Pi gradients between the matrix and mitochondrial intermembrane space [88]. In the late version of the model [89] the ATP/ADP ratios in the matrix and activity of ATP synthase are dependent on ΔΨ, the electric component of mitochondrial membrane potential. This version of the model employs the complete mathematical model of the Pi carrier based on the probability approach, allowing predicting the dynamics of Pi accumulation in the matrix in exchange for matrix OH^−^ ions at the expense of mitochondrial proton-motive force, ΔpH [89]. Mitochondrial oxidative phosphorylation is activated by ADP and Pi produced from ATP hydrolysis by myosin in the myofibril compartment. The kinetics of ATP hydrolysis by myosin in contracting muscle was predicted from d*P*/d*t* change in isovolumic rat heart: a linear increase in ATP hydrolysis rate up to 30 ms, followed by its linear decrease to zero at the 60-th ms of contraction-relaxation cycle. The total duration of this cycle was taken to be 180 ms [88]. The model considers CK compartmentation and the real non-equilibrium kinetics of the creatine kinase reactions in different cellular compartments [88,89]. The molecules of cytoplasmic isoenzyme of CK (MM-CK) are distributed in the myofibrillar and cytoplasmic spaces (Figure 5) and transphosphorylate ADP to ATP at the expense of PCr utilization. This direction of *in vivo* functioning is favored by intrinsic thermodynamic parameters of MM-CK. A part of cellular CK, 31% of its total activity, is localized in the mitochondrial compartment. In mitochondria, this isoenzyme of CK (MtCK) is tightly anchored to ATP/ADP translocase and outer surface of inner mitochondrial membrane by cardiolipin molecules [95]. The resulting close proximity of MtCK and translocase allows direct tunneling of adenine nucleotides between their adjacent active centers; this tunneling is the actual base for shifting the MtCK reaction toward the synthesis of PCr from translocase-supplied ATP [2,3,14,15,17–24].

Mathematical modeling of cellular CK shuttle became possible only after special modeling of the kinetics of mitochondrial ATP/ADP translocase by the probability approach and of functional coupling of translocase with MtCK [96–98]. In both versions of the model [88,89] functional coupling of MtCK to translocase was simulated by means of dynamically changing high local ATP concentrations in a 10-nm narrow space (microcompartment) between coupled molecules. This simplified approach was used because of a large number of calculations in original precious probability model of coupling. The probability model was used to check the validity of calculations in a simplified approach. The compartments of the system communicate by metabolite diffusion along the radii of myofibrils and mitochondria (Figure 5). The diffusion of ADP and ATP through mitochondrial outer membrane is restricted due to VDAC molecular complexes within MI [20,21,24]. The computations of diffusion and chemical events were performed for every segment of diffusion path at each 0.01 ms time step [88]. This allows the simulation of the space-dependent changes throughout the entire cardiac cycle.

This model was used for modeling the data by Williamson *et al*. [99], who used the modified working heart protocol of Neely for studies of the bioenergetics of perfused rat heart under a number of conditions of substrate availability at increasing workloads. Working heart protocol is the only physiological experimental method which allows reproducing on the isolated heart the Frank-Starling phenomenon—Increase of heart work and oxygen consumption by increasing left ventricle filling [99].

In our modeling, we consider only the data of [99] for normoxic perfusion in the presence of glucose-octanoate as substrates, when the workload was increased to its maximal value as high as 174 μmol O_2_/min/g dry mass. Actually, authors [99] determined and presented all parameters necessary for simulation with our model [89], except for total Cr contents in rat hearts. Comparative analysis of papers [99] and [100] gave the total Cr content of 73.7 μmol/g dwt; we used the value of 73 μmol/g dwt similar to that used in our work [89]. Phosphate in ATP + ADP + AMP + PCr + Pi were measured as 131.5 and 135.8 μmol/g dwt [100]; we used the value of 135 μmol/g dwt. ATP + ADP was measured as 24.8 μmol/g dwt [99]; we used the value of 25 μmol/g dwt. Details of these calculations can be found in two our papers, [101] and [89]. These data can be used for calculation of molar concentrations of metabolites. With taken dry mass content in idealized perfused rat heart, 202.6 g/kg dwm we will have 25 × 202.6 = 5065 μmol, or 5.065 mmol of adeninenucleotide (ADN) contents in 1 kg of heart tissue. With 134.6 mL of mitochondrial matrix water per kg wm and 394.4 mL of extra-matrix water space for free diffusion of metabolites per kg wm, an average concentration of ADN will be 5.065 mmol of ADN/(0.1346 + 0.3944) L of cell water, or 9.57 mM. This value is about twice higher than the ADN content in these hearts, 5.065 mmol/kg wm.

Only one set of parameters was changed in this model, the maximal velocities of cellular CK reactions in the reverse direction (ATP production) were increased 1.5-fold to attain 64 mmol/s/kg wm. This value matches the maximum total activity of CK in the direction of MgATP synthesis, 62.4 ± 4.5 mM/s, measured in perfused rat hearts [48]. The concentrations of metabolites in perfused hearts were predicted using our concept of “idealized perfused heart” [101]. According to this concept, idealized perfused heart contains 202.6 g of dry mass per kg of its wet mass; metabolites in these hearts are distributed in 134.6 mL of mitochondrial matrix water and in 394.4 mL of extra-matrix water space for free diffusion of metabolites [101]. To make easier the comparison of modeled data of [99] with our modeled data in [89], we recalculated the data in [89] for 202.6 g of dry mass contents and 1.5-fold enhanced maximal activities of cellular CK enzymes. Other conditions of simulations are the same as used in [89].

**Figure 6:**
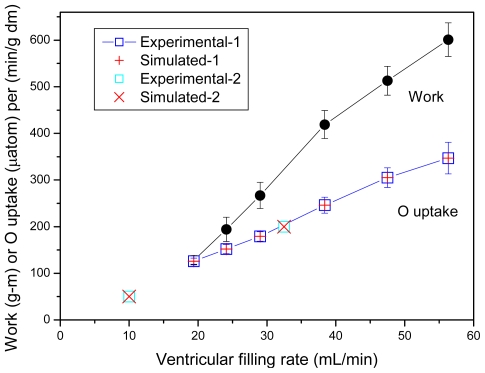
Simulation of experimental data of Williamson *et al*. [99] on oxygen consumption rates by isolated rat hearts perfused according to Neely’s procedure. Data marked as “Experimental-1” were taken from Figures 5–6 [99], and marked as “Experimental-2”—From Figures 7 and 8 [99]. Points marked as “Simulated-1” and “Simulated-2” correspond to modeled “Experimental-1” and “Experimental-2” data, respectively. Hearts were perfused in media with 5 mM glucose, 10^−2^ U/mL of insulin, and 0.5 mM octanoate (Experimental-1), or with 1 mM pyruvate (Experimental-2). Data on the work output (black circles) are included to demonstrate the correspondence between oxygen uptake and work output at their extreme values.

**Figure 7:**
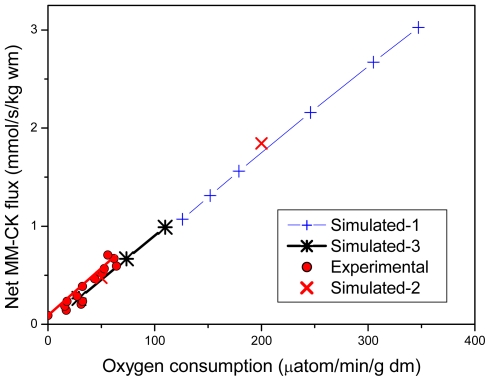
Comparison of simulated net fluxes through cellular MM-CK system with experimental data of [99]. Simulation data actually continue experimental ones. At a highest workload, the net flux through MM-CK amounts to 89.2% of total flux through ATP-synthase. Curves marked as “Simulated-1” and “Simulated-2” correspond to those in Figure 6. Simulation data for the curve, marked as “Simulated-3” were taken from Table 5 in [89], lines “Workstate transitions with pyruvate”. Data, marked as “Experimental” were taken from [9].

Figure 6 reproduces experimental data from Willamson *et al*. [99] and shows that our model fits the experimentally determined values of respiration rates. Figure 7 shows that the creatine kinase flux is increased linearly with increase of the rate of respiration.

**Figure 8:**
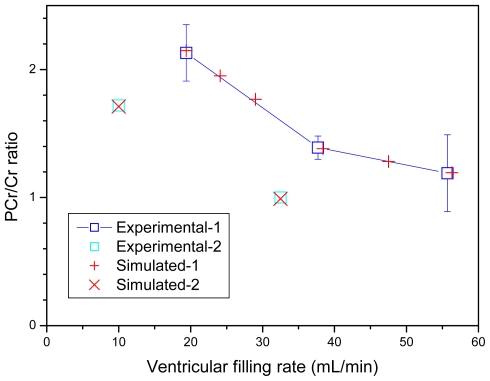
Simulation of experimental data of Williamson *et al*. [99] on PCr/Cr ratios in perfused rat hearts. The symbols and designations are the same as in Figure 6.

Model also reproduces the observed PCr levels at different workloads (Figure 8).

**Figure 9:**
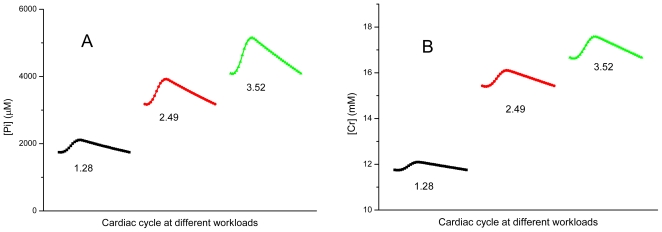

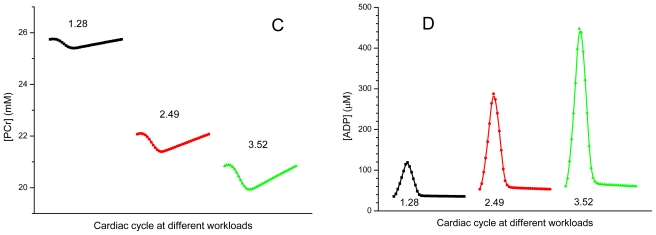
Simulated dynamics of Pi (**A**), creatine (**B**) and PCr (**C**) concentrations in the core of myofibril at three levels of workload; (**D**) Simulated dynamics of ADP concentrations in the core of myofibril at three levels of workload. Workload levels are indicated in terms of ATP synthesis by mitochondria, mmol/s/kg wet mass.

Figure 9 demonstrates the calculated changes of metabolites—Pi, Cr and PCr within contraction cycle at different workloads, and resulting cycling changes in ADP concentrations in the myofibrils’ core. These transient changes in the ADP concentration are caused by release of ADP by ATPases and its rapid rephosphorylation by myofibrillar creatine kinase ([88], see Figure 3B). These signals are transmitted together with changes in AMP concentration to mitochondria (Figure 3C).

**Figure 10:**
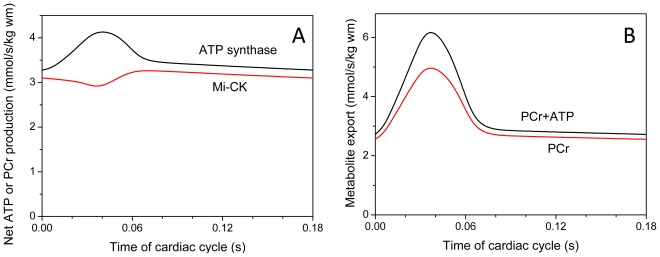
(**A**) Dynamics of net ATP production by ATP-synthase and net PCr production by Mti-CK within contraction cycle at maximal workload, 3.52 mmol/s/kg wm. Net PCr production by MtCK is 89.3% of ATP-synthase job; (**B**) Dynamics of metabolite export from mitochondria at maximal workload, 3.52 mmol/s/kg wm. Direct ATP export amounts to 10.6% of total energy export from mitochondria

**Figure 11:**
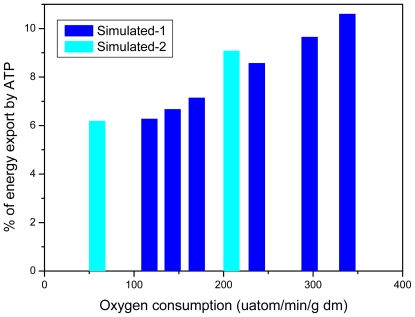
Modeled low proportions of direct energy export from mitochondria by ATP in experiments of [99]. The symbols and designations are the same as in Figure 6.

Figure 10 shows the calculated rates of mitochondrial ATP synthesis and PCr production (A) and metabolite export into cytoplasm (B). This Figure also shows that about 90% of energy flux is carried by PCr, and Figure 11 shows that this is valid for any workload and rate of oxygen consumption. This is in good concord with experimental data of flux determination by Dzeja *et al*. described above [4–8].

#### 1.4.2. Multiscale “Sloppy” Modeling of CK Fluxes by Hetting—van Beek

**Figure 12:**
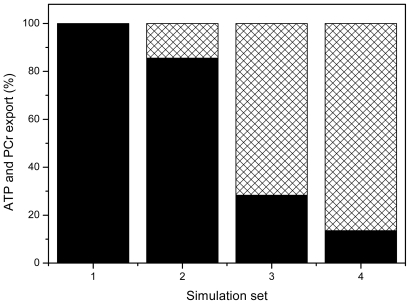
Figure presents calculated by model [89] proportions of ATP (black areas) and PCr (hatched areas) export by mitochondria in contracting rat cardiac cells at a high workload. These data were mainly presented in our review in 2007 [104]. While in system 1 without CK (column 1) the energy from mitochondria is exported completely, as expected, by ATP molecules, in the system 2 with free (uncoupled) CK and without diffusion limitations on MOM a small part, about 15%, of energy export is carried by PCr molecules (column 2). Situation dramatically changes, if the system 2 is upgraded to include local coupling of MtCK to ANT—In this system 3 energy export by PCr molecules rises up to about 72% of total energy export (column 3). Finally, in the complete system, which includes both CK to ANT coupling and diffusion restrictions for ADP on MOM, the energy export by PCr is prevailing, up to about 87% of total energy export (column 4).

The A-S model was upgraded further by Vendelin *et al*. for 2- dimensional analysis of metabolites’ diffusion within ICEUs [102]. The new model called VAS model is a clone of the original model of Aliev and Saks, 1997 [88]. Both models, and our later modification [89], explain the predominant, up to 90–95%, energy export from mitochondria by PCr molecules basing on joint action of two main mechanisms, (a) local functional coupling of mitochondrial CK (MtCK) to mitochondrial adenine nucleotide translocase (ANT), and (b) severe diffusion restrictions for ADP (and ATP) on mitochondrial outer membrane (MOM). Hetting and van Beek declare that they have used this model to find only 15% of energy leaving mitochondria by the PCr pathway [90,103]. Now the question arises as to what the reasons for such a discrepancy may be and why the outcome with the multiscale sloppy model differs so much from data obtained with our previously reliable model? The answer becomes very evident when one analyses which parameters are important in the model and which manipulations led the authors to this failure. Figure 12 presents calculated by model [89] proportions of ATP (black areas) and PCr (hatched areas) export by mitochondria in contracting rat cardiac cells at a high workload.

These data were mainly presented in our review in 2007 [104]. While in system 1 without CK (column 1) the energy from mitochondria is exported completely, as expected, by ATP molecules, in the system 2 with free (uncoupled) CK and without diffusion limitations on MOM a small part, about 15%, of energy export is carried by PCr molecules (column 2). Situation dramatically changes, if the system 2 is upgraded to include local coupling of mitochondrial CK to ANT—In this system 3 energy export by PCr molecules rises up to about 72% of total energy export (column 3). And finally, in the complete system, which includes both CK to ANT coupling and diffusion restrictions for ADP on MOM, the energy export by PCr is prevailing, up to about 87% of total energy export (column 4). In contrast, simulations in [90] indicate a very low, 15 ± 8%, proportion of energy export by PCr molecules. Such a low proportion is just characteristic for the system with free, uncoupled MtCK and without diffusion restrictions for ADP on MOM, according to our simulations (Figure 12, system 2). Further, the authors manipulated with the parameters of the model, taking the maximal rate of the MtCK reaction to be only 50% of that of ATP synthase, while experiments show that these rates are equal [105]. In this way, by incorrect selection of model parameters they already programmed the failure to describe the experimental data correctly. This coincidence is not occasional, as authors in [90] indeed neglected MtCK to ANT coupling; diffusion restrictions for ADP on MOM were small, with membrane permeability parameter PS_m_ of 31.7 s^−1^, in contrast to that value in [102], 0.1 s^−1^. What are the declared reasons for these simplifications? An early observations of Erickson-Viitanen *et al*. 1982 [106], that coupling strength may be very low for > 0.7 mM ATP, lead van Beek to proposal, that MtCK to ANT coupling can be neglected at physiological, millimolar, ATP levels. In the same year Jacobus and Saks presented the paper on extensive research of MtCK to ANT local coupling in isolated rat heart mitochondria, exploring practically all possible regimens of system functioning [105]. Later, in 1993–1996, Aliev and Saks have described these relations in the frameworks of original so called “probability” model [96–98]. The model, well fitting the data of Jacobus and Saks [105], indeed confirmed the decrease in control strength of coupling with the rise in ATP concentrations for the case of direct transfer of ATP from ANT to MtCK in isolated mitochondria [107]. But is such a decrease enough for completely neglecting the coupling phenomenon at millimolar ATP levels in the cells *in vivo*? Using isolated mitochondria, it was shown that by simple addition of creatine to a mitochondrial suspension, in the presence of respiratory substrates without added nucleotide, these mitochondria produce and release PCr into the supernatant that is formed by intra-mitochondrially cycling ATP and ADP via creatine-stimulated respiration (see Figure 4 in [108]), indicating a strong coupling between respiration, ATP-synthesis, ATP export through the inner mitochondrial membrane, transphosphorylation of ATP to PCr by mitochondrial CK in the intermembrane space and export of PCr through the outer membrane. The efficiency of this coupling is very significantly increased due to cytoskeletal-mitochondrial interactions in the cells *in vivo* [2,3,9,17–24]. In their pioneering studies Frank Gellerich and Dieter Brdiczka [109–112] already pointed out the importance of accounting for possible limitations of ADP and ATP diffusion across the mitochondrial outer membrane. This proposal has been found to be true for the cardiac cells *in vivo* [17–24,113–117]. 20 years of research in many laboratories have shown that mitochondrial behavior *in vitro* and in permeabilized cells *in situ* are very different [17–24,113–117]. Thus, in the latter case the affinity of mitochondrial oxidative phosphorylation for exogenous ADP is significantly decreased due to interaction of mitochondria in the cells with cytoskeletal components as tubulin and plectin, in particular with tubulin beta II [17–24]. Association of βII-tubulin with VDAC and its coexpression with MtCK has fundamental consequences for the regulation of metabolite and energy fluxes between mitochondria and cytoplasm in cardiac cells. These proteins were supposed to form a supercomplex, the Mitochondrial Interactosome (MI) in contact sites of the inner and outer mitochondrial membranes [17–24]. The MI supercomplex includes βII tubulin, VDAC, MtCK and ATPsynthasome, consisting of structurally bound ATPsynthase, ANT and PIC (Figure 2). In the cristae membranes, there are only functionally coupled MtCK and ATP synthasome. The latter system is also present in isolated mitochondria which have lost tubulin and therefore VDAC permeability is high (low apparent KmADP). In the MI the permeability of VDAC for adenine nucleotides is specifically restricted: kinetic studies of MtCK within MI showed that its apparent dissociation constant for extramitochondrial MgATP, K_a_, is increased about 200 times, up to 2.04 mM, in comparison with *in vitro* value, 0.016 mM [20]. Martin Picard, Tanja Taivassalo and their coworkers [118,119] have recently shown that mitochondrial isolation induces fragmented organelle morphology, dramatically sensitizes the permeability transition pore sensitivity to a Ca^2+^, dramatically increases H_2_O_2_ production. These alterations are qualitatively similar to the changes in mitochondrial structure and function observed *in vivo* after cellular stress-induced mitochondrial fragmentation, but are generally of much greater magnitude. Furthermore, mitochondrial isolation markedly altered electron transport chain protein stoichiometry [118,119].

Further, experimental studies with application of Metabolic Control Analysis showed very high control strength within the MI structure at high level of ATP in the permeabilized cardiomyocytes [76,77]. This conclusion is in concord with the results of calculations shown below. Table 1 presents the data of modeling of MtCK to ANT coupling at high ATP levels in medium using our probability model [97,98]. Cr levels were constant, 10 mM, ADP and PCr levels were taken to be zero, and PCr/O_2_ ratios was calculated at ATP/O_2_ ratio of 6.0. The model simulations show that even at 10 mM ATP levels in medium the stimulation of oxidative phosphorylation by MtCK is very substantial. In the same system, without coupling the stimulation of CK is about the same, while the oxidative phosphorylation activation is negligible, as expected (data not shown). The results of probability model calculations are essentially in line with recent direct experimental measurements by Timokhina *et al*. [75]. Basing both on model simulation and experimental data, we can conclude that an assumption of van Beek [103] on nullifying the MtCK to ANT coupling at millimolar ATP levels cannot be regarded as justified, since they have not accounted for the differences in mitochondrial behavior *in vitro* and *in vivo*.

Very high permeability of MOM to ADP was inferred by van Beek [90,103] from the model fitting of his original experimental data on mitochondrial response times, t_mito_. With taken general model parameters, the experimental t_mito_ values of about 4 seconds were fitted by membrane permeability parameter PS_m_ = 13.3 s^−1^, while with PS_m_ = 0.1 s^−1^ from [102] the t_mito_ was about 15 s (Figure 3 in [103]), too far from experimental determinations. In recent paper PS_m_ value to fit experimental data was estimated as even higher, 31.7 s^−1^ [90]. In the frameworks of this logic, t_mito_ values in basic models from the group of Saks (so named VAS (Vendelin-Aliev-Saks) models [102]) should be high, far from experimental values.

**Figure 13:**
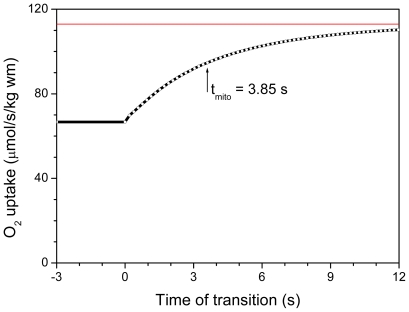
Model-calculated time course of increase in oxygen uptake rate during transition from low (0.400 mmol ATP*s^−1^*kg wm^−1^) to medium (0.678 mmol ATP*s^−1^*kg wm^−1^) workload. Steady-state parameters for these workloads are indicated in Table 3 in [89] for glucose-perfused rat hearts.

Figure 13 demonstrates our model-calculated time course of increase in oxygen uptake rate during transition from low (0.400 mmol ATP*s^−1^*kg wm^−1^) to medium (0.678 mmol ATP*s^−1^*kg wm^−1^) workload. Steady-state parameters for these workloads are indicated in Table 3 in [89] for glucose-perfused rat hearts. Data of Figure 13 clearly indicate that in the system with local MtCK to ANT coupling experimental values of t_mito_ can be approximated even at imposed severe diffusion restrictions for ADP on MOM. The parent relation of our models [88,89] to a model used by van Beek *et al*. [90,103] allows us to repeat the simulations of van Beek *et al*. in order to understand how is it possible to use similar models to attain principally different results. Data of such modeling, performed with our model [89], are listed in Table 2. First line of Table 2 illustrates the data in Figure 13: our complete system with MtCK tightly coupled to ANT and with severe restrictions for ATP/ADP diffusion on MOM can predict reliable t_mito_ value at very high, about 90%, fraction of PCr diffusion out the rat heart mitochondria. Myoplasmic PCr/Cr ratios during the diastole are high, 2.6–1.8. In the System A on the second line in Table 2, the basic parameters of our model were changed toward that’s in [90,103]: coupling of MtCK to ANT was completely omitted, the fractional ratio of MtCK activity to total cellular CK activity was decreased from 31.25% [88] to 8%, according to [90] (7.2%) and [103] (8.9%). The MOM permeability to ATP/ADP was kept high.

In such system t_mito_ value is high, 8.7 s, out the experimental estimations. In similar conditions van Beek estimated t_mito_ as high as 15 s (Figure 3 in [103]). Please, note decreased PCr/Cr ratios and rather high fraction of PCr export from mitochondria based on high diffusion restrictions on MOM.

High t_mito_ values in System A-type simulations permitted van Beek to lower MOM permeability to obtain t_mito_ about 4 s [103]. This result can be reproduced by our model on decreasing the MOM permeability restriction coefficient from 0.007 to 0.1 (Third line in Table 1). Please, note the dramatic decrease in PCr export percentage, 19–24%, close to that’s published by [90]—15 ± 8%. With impaired MOM influence the PCr/Cr ratios were restored up to values for complete system. Another difference in simulations of van Beek *et al*. was decreased cellular contents of total creatine (Cr + PCr) and adenine nucleotides. Last line in Table 1 repeats simulations of previous one, but with 1.7-fold decreased contents of adenine nucleotides and total creatine and 2.6-fold decreased contents of Pi, according to Table 2 in [103]. This manipulation further decreases t_mito_ and PCr/Cr ratios, not affecting the low fraction of energy export by PCr. The question arises, how justified are the changes in model parameters made in van Beek’s group? Authors ascribe these changes to peculiarities of rabbit heart muscle [103]. Available data, however, do not support this viewpoint

Low ratios of MtCK activities to total cellular CK activity were predicted by [90,103] on the basis of MtCK activities per unit of mitochondrial mass in relation to total CK activity. But these parameters are essentially similar both for rat and rabbit hearts [120]. Specific cytochrom aa_3_ contents are slightly lower in rabbit heart mitochondria (0.353 ± 0.07 nmol/mg protein [121]) than in rat ones (0.445 ± 0.09 nmol/mg protein [121] ), but the molecular ratios of MtCK and aa_3_ are similar (2.43 ± 0.26 and 2.35 ± 0.25 mol MtCK/mol aa_3_ for rabbit and rat heart mitochondria, respectively [121]). Taking into account very close morphometry estimates for mitochondrial contents in rat and rabbit hearts (32.03 ± 1.83% and 28.86 ± 1.01% of cell volume, respectively [122]), we have no valid basis whatsoever for assuming a manifold differences in total MtCK activities and contents in these muscles. Our estimates of relative MtCK contents and activities in rat hearts, 31%, were based mainly on direct biochemical measurements of CK isoenzyme distributions in rat hearts [123]. As related to decreased cellular contents of total creatine in rabbit hearts [90,103], their data do not correspond, for example, to data of Weiss *et al*. [124]: in rabbit hearts PCr and Cr contents were measured as 66.2 ± 14.7 and 26.2 ± 4.5 μmol/g dm, respectively [124]. This estimate is even higher than the value of 73 μmol/g dm used in our model [89]. Based on the facts considered, we can conclude that assumptions in papers [90,103] by the authors on very low ratios of MtCK activity to total cell CK activity as well as on decreased total creatine contents used for rabbit hearts are rather erroneous. Correspondingly, the conclusion on a rather high permeability of MOM to ATP/ADP made on the basis of such model parameters, again, cannot be regarded as justified, since experimental evidence shows the contrary to be true.

#### 1.4.3. PCr Fluxes Lost: The Vendelin-Hoerter’s Model

A recent manuscript published in the Journal of Biological Chemistry by Vendelin, Hoerter, Mateo, Soboll, Gillet and Mazet entitled: “Modulation of energy transfer pathways between mitochondria and myofibrils by changes in performance of perfused heart” [91] is also dealing with important questions of energy flux in the perfused heart. According to the new results by Vendelin *et al*., the stability of total CK unidirectional flux is lost at extremely high energy demand levels leading to a drop of total CK unidirectional flux and to a bypass of CK shuttle by direct ATP transfer from the mitochondria to the myofibrils [91]. For treatment of their data they used a model that could be called Vendelin-Hoerter model [91].

These results and their interpretation, made in the work referred to above, are not consistent with a large body of existing data, including Vendelin’s own results published before in many articles [9,102,104]. They raise many questions among specialists in the field that worked on the very question of how in cardiac muscle cells energy is transferred from mitochondria to the contractile apparatus, ultimately supporting cardiac muscle contraction. In the increasingly important field of metabolic research in the area of Systems Biology [27], the principal and most precise experimental approach to the *in vivo* kinetic studies and metabolic flux determination is the isotope tracer method, as described above. Another technique of labeling the phosphoryl groups in ATP and PCr in heart cells is ^31^P NMR saturation or inversion transfer, already applied in many laboratories [1,28,47–50]. These are, however, indirect methods and subject to some possible problem with NMR invisible pools as indicated above. Nevertheless, when correctly used and analyzed, these methods also allow revealing the increase of CK flux registered by isotope tracer method described above, since they measure the total unidirectional fluxes between ATP and PCr catalyzed by CK in all cellular compartments. Under conditions of metabolic stability, when PCr content does not change, an increase in the rate of PCr production in one compartment equals an increase of PCr utilization for local ATP generation in another compartment. Indeed, Bittle and Ingwall [49] and Kupriyanov *et al*. [50] have succeeded in showing the increase of CK flux with increase of workload of the heart, well fitting with the results of Dzeja *et al*. [4–9]. In several laboratories it was found that CK flux was independent of workload, which was varied, however, only in limited range, probably too small to draw clear conclusions.

The CK pathway is now described in great detail [9–28]. In heart cells, net reaction rates of mitochondrial CK (MtCK) and MM-CK in myofibrils function in opposite direction and increase with workload (Figure 3A,B). In the cells *in vivo*, tubulin binding to voltage-dependent anion channel in mitochondrial outer membrane specifically decreases its permeability for ATP, but not for creatine and phosphocreatine and coupled reactions in Mitochondrial Interactosome, consisting of tubulin, VDAC, MtCK and ATP Synthasome result in effective phosphocreatine (PCr) synthesis with PCr/O_2_ ratio close to 6 [75]. This leaves little room for direct transfer of ATP and is consistent with ^18^O_2_ measurements described above. There is, however, also a certain proportion of MM-CK in the cytoplasm that is in a quasi equilibrium state, which does not depend on the workload, if PCr and ATP contents do not change, as it is seen in heart in the state of metabolic stability [125]. Even if all CK would be in a quasi-equilibrium state, the CK fluxes measured by ^31^P-NMR saturation or inversion transfer techniques would still show the total CK activity that is present. If the cells are intact, this activity would not change, and CK flux measured by ^31^P NMR therefore should not change either and never decrease. Vendelin *et al*. [91] now are the first observing a dramatic decrease of total unidirectional CK flux by factor of 2 by increasing the workload artificially to extreme values (Figure 8 in their manuscript). According to these data, 50% of CK flux was lost, and no mention of adenylate kinase or glycolytic fluxes were made. Where then all these fluxes are gone [126]? For the authors, this is evidence for “direct ATP transfer”, but the underlying molecular events are not further discussed. In our opinion, a loss of 50% of the CK flux may reflect a dramatic loss of total CK activity present in the cells, with contributions of AK and GL systems not taken into account.

**Figure 14:**
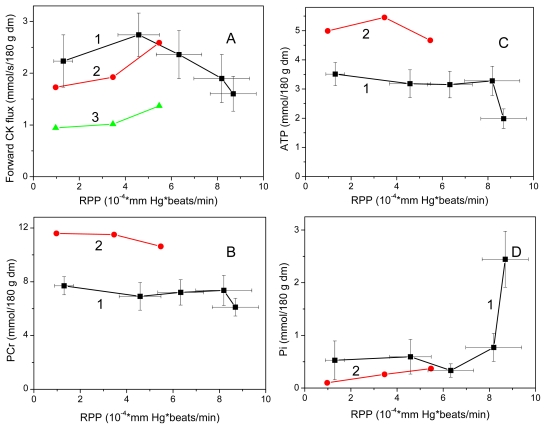
Metabolic disaster induced by catecholamines in Langendorff-perfused rat hearts by Vendelin *et al*. [91]. Workload dependence of main metabolic parameters, forward CK flux (**A**), and PCr (**B**), ATP (**C**), Pi (**D**) contents in pyruvate, 10 mM, perfused isolated rat hearts, in papers of Vendelin *et al*., 2010 (Curves 1). For comparison, the data by Dos Santos *et al*., 2000 [89] are given (Curves 2,3). Curves 2 display the experimental (**B**,**C**) and modeled (**A**,**D**) data in [89]. Curve 3 indicates the modeled unidirectional forward CK flux through MM-CK [89]. Data of [89] are without statistical deviations as there were taken mainly from the Tables 3 and 4 with basic data for mathematical modeling.

The reason for such a loss of CK activity may be found in method used: the combination of increased Ca^2+^ concentration together with isoprenaline, a method which is known to induce severe damage in cardiac cells [127]. This effect is called catecholamine-induced necrosis of the heart tissue, this discovery led to development of β-blockers for heart protection [46]. Increase of intracellular Ca^2+^ concentration induced by catecholamines is known to induce mitochondrial permeability transition pore (PTP) opening, mitochondrial swelling, MtCK detachment, sarcolemmal rupture and CK release. It is also known that uncoupling of MtCK from the mitochondrial adenosine nucleotide translocase (ANT), which results in a loss of creatine-stimulated respiration, ultimately leads to significantly increased production of highly reactive ROS or RNS species [128–131]. CK, which shows exquisitely high sensitivity to ROS and RNS, has been shown to get inactivated preferentially already at very low concentrations of these free radicals, and MtCK octamers were shown to fall apart into dimers that are no longer able to bind to the mitochondrial inner membrane [131]. This conclusion is directly confirmed by authors own data shown in Table 2 in the paper by Vendelin *et al*.: total content of ATP decreased from 7.78 to 4.7 mM in the presence of high Ca and isoprenaline. The dramatic 40% decrease of ATP and 200% increase of Pi (see Figure 14) are clear signs of metabolic disturbances and catecholamine—induced myopathy that is never seen in normal cells. Thus, what is cited in the paper as “extreme workload conditions” rather corresponds to a pathological state where CK is likely to be inactivated by mitochondrially generated ROS and released from the mitochondrial membrane, rendering the enzyme incapacitated for its normal physiological function for PCr mediated energy transfer.

In Figure 14 the data of Vendelin *et al*. [91], obtained from rat heart perfusion in solutions with 0.5, 1.8, 4.0 mM Ca, 0.5 mM Ca plus Isoprenaline and 4.0 mM Ca plus Isoprenaline (that is a sequence of the increase in Rate Pressure Product, RPP), are compared with the data [89] from rat heart perfusion in solutions with 0.5, 1.25, 3.5 mM Ca. As in [89] the data for the sake of comparison were given for 180 g dry mass of idealized heart, the data of Vendelin *et al*., 2010 were recalculated for the same units, simply multiplying them by the coefficient 0.4216. This coefficient takes into account the water/protein ratio of 435.2 mL H_2_O/kg wm in Vendelin *et al*., 2010, and small differences in used values of protein contents (160 g protein/kg wm in Vendelin *et al*., 2010 and 155 g protein/kg wm in [89]).

Note that with this drastic means of catecholamine-induced increase in workload in the presence of 4.0 mM Ca leading to degradation of 40% of ATP, manifold increase of Pi content and loss of CK flux, respiration rate achieved in the work by Vendelin *et al*. is equal to 75 μmol O_2_ per min per g dry weight, less than half of the maximal respiration rate 168 μmol O_2_ per min per g dry weight obtained in experiments with working heart model when workload change is induced by changing ventricular filling on the basis of Frank-Starling mechanism [9,99].

Most surprisingly, the authors themselves were aware of the importance of the stability of the metabolites’ levels in the studies of energy fluxes: in their previous work [132] they write correctly that “the stability of the preparation was checked by comparing fully relaxed control spectra (repetition time 10 s) acquired before and after the magnetization transfer experiment; any heart showing more than 10% variation in its metabolite content was discarded”. That means that according the authors own criteria the data published in [91] are not correct; in the work with stable ATP levels they were able to detect easily the PCr flux between mitochondria and cytoplasm [132].

The other main question is: how adequate are the “mathematical models” used. Figure 9 in their work indicates that at conditions of normal perfusion with 1.8 mM Ca, when the hearts are metabolically quite stable, the fluxes can be well modeled by three (!) model schemes with the share of direct ATP export from 0 to 100%. In other words, such fitting may give any result needed for any purposes! It is very unfortunate that the authors did not discuss all available literature and made proper controls in an attempt to refute obvious arguments as stated above. For example, they could provide evidence of reversibility of the measured phenomenon. If their interpretation were correct, the loss of CK-mediated energy transfer seen under extreme workload should be reversible, that is, CK-mediated flux in hearts first exposed to extreme workload should reappear again under subsequent exposure to lower work-load. Thus, our contention that the CK system including the mitochondrial integrity was severely and irreversibly damaged by the conditions of perfusion with catecholamines at high workloads cannot be refuted and thus no firm conclusion can be drawn about the energy flux distribution in these experiments. The results can give only some information of changes in CK flux in a pathological state, particularly in catecholamine-induced cardiomyopathy.

In conclusion, both correct modeling by taking into account all existing experimental data, as well as qualified experiments avoiding artifacts such as induced in the work by Vendelin *et al*. [91], are needed to accurately describe in a most realistic way the energy fluxes in the heart both in health and in pathology.

## 2. Conclusions

There is an excellent agreement between recently published data from many laboratories, which give now the possibility of quantitative description of the energy fluxes between mitochondria and cytoplasm in muscle cells. By using the ^31^P saturation transfer spectroscopy to study the kinetics of the creatine kinase in muscle cells by saturating γ-ATP phosphate and recording the transfer of magnetization to PCr, Nabuurs *et al*. discovered the binding of ATP and ADP to macromolecular complexes in the cells, explaining the mechanisms of ATP compartmentation and non-equilibrium state of the creatine kinase reaction in the cells [1]. Fundamental studies by Goldberg, Dzeja and Terzic groups have shown by using ^18^O transfer method that in the heart, the phosphocreatine fluxes from mitochondria into cytoplasm transport about 80% of energy needed for contraction and ion transport, and about 20% of energy is transported into cytoplasm via adenylate kinase and glycolytic phosphotransfer pathways [4–9]. Very similar results have been obtained by studies of the creatine kinase reaction in the permeabilized cardiac cells and by computer analysis with the use of the mathematical model of compartmentalized energy transfer [88,89]. The failures of several models to describe experimental data published in literature are due to incorrect model parameters selected (Hetting and van Beek) [90] and by choosing non-suitable experimental models (Vendelin and Hoerter) [91].

## Figures and Tables

**Table 1:** Probability model [97,98] calculations of ATP synthesis and PCr production rates in mitochondria with coupled CK/ANT system at high ATP levels in medium.

[ATP], mM	MtCK Rate (PCr Production), % of Maximum	ANT Rate (ATP Export), % of Maximum	PCr/O_2_
1	62.61	53.55	7.01
2	63.95	52.12	7.36
5	64.83	46.19	8.44
10	65.17	38.22	10.2

**Table 2:** Model [89] calculations of t_mito_, myoplasmic PCr/Cr ratios and energy export by PCr from mitochondria on workstate transitions from low to medium workloads in different configurations of heart cell energetics.

System Description	t_mito_, s	Myoplasmic PCr/Cr in Diastole		Energy Export by PCr, %	

		Before Transition	After Transition	Before Transition	After Transition
System complete with MtCK tightly coupled to ANT and severe restrictions for ATP/ADP diffusion on MOM	3.9	2.64	1.82	91.4	90.0
System A with low MtCK activity with no coupling, but maximal restrictions on MOM	8.7	1.82	0.88	73.2	62.7
System with no coupling and weak restrictions on MOM	4.4	2.53	1.68	23.5	19.4
System with no coupling, weak restrictions on MOM and reduced metabolite contents	2.9	1.96	1.28	23.3	19.4
